# Comparison of clinical outcomes of supercapsular percutaneously-assisted approach total hip arthroplasty versus conventional posterior approach for total hip arthroplasty in adults: a systematic review and meta-analysis

**DOI:** 10.1186/s12891-023-07126-x

**Published:** 2024-01-02

**Authors:** Yize Zhao, Wenchen Sun, Chen Wang, Xinyi Xie, Ganjun Feng

**Affiliations:** 1 Department of Orthopedic Surgery and Orthopedic Research Institute, West China Hospital, Sichuan University, Chengdu, Sichuan China; 2 Anhui Medical University, Hefei, Anhui China

**Keywords:** Supercapsular percutaneously-assisted approach total hip, SuperPATH, Posterior lateral approach, Posterior approach, Total hip arthroplasty

## Abstract

**Objective:**

This meta-analysis was aimed to compare the postoperative clinical outcomes between the supercapsular percutaneously assisted total hip (SuperPATH, SP) and conventional posterior/posterolateral approach (PA) for total hip arthroplasty in patients who have failed conservative treatment for hip-related disorders.

**Methods:**

PRISMAP guidelines were followed in this systematic review. CNKI, Wanfang, PubMed, Embase, Cochrane, Web of Science databases and the reference list grey literature were searched for studies according to the search strategy. Endnote (version 20) was used to screen the searched studies according to the inclusion and exclusion criterias and extract the data from the eligible studied. RR and 95% CI were used for dichotomous variables and MD and 95% CI were used for continuous variables. All analyses and heterogeneity of outcomes were analysed by Review Manage (version 5.4). Publication bias of included studies was analysed by Stata (version 16.0).

**Results:**

Thirty-six randomized control studies were included. Compared to PA group, SP group had a shorter incision length, less intraoperative blood loss, a shorter length of hospital stay and do activities earlier. Hip function (HHS) was significantly improved within three months postoperatively. Pain of hip (VAS) was significantly reduced within one month postoperatively. The state of daily living (BI) was significantly improved within three months. Patients' overall health status (SF-36) improved significantly postoperatively. There was no difference in postoperative complications between the two approaches. PA had a shorter operative time and a higher accuracy of prosthesis placement.

**Conclusion:**

The advantages of SuperPATH include accelerated functional recovery and less trauma associated with surgery. However, it required a longer operative time and implantation of the prosthesis was less accurate than that of PA.

**Supplementary Information:**

The online version contains supplementary material available at 10.1186/s12891-023-07126-x.

## Introduction

Total hip arthroplasty (THA) is known as one of the most successful surgical procedures of the last half century [1] and is widely used for the treatment of various hip diseases that failed conservative treatments or advanced hip disease [2], due to its effectiveness in reducing pain, correcting deformity, improving hip function, and low postoperative revision rates, it improves the quality of life of patients significantly [3]. From 2012 to 2019, the average annual growth rate of total hip replacement surgery performed in China was 8.56% [4]. In 2018, the number of PA performed in China reached 400,000 [5].

The conventional posterolateral/posterior approach(PA) has good and definite efficacies and is the most widely used way for ease of manipulation, clear intraoperative visual field exposure, and stable postoperative outcomes [6]. However, it is not compatible with today's requirements and expectations of rapid rehabilitation with more precise, accurate, safe, and less invasive [7]. The supercapsular percutaneously assisted total hip (SuperPATH, SP) is an emerging THA approach, which is a minimally invasive surgical approach based on the posterolateral approach. The minimally invasive procedure has the advantage of reducing infection, dislocation, intraoperative bleeding, speeding recovery [8], and SP does not require cutting the muscles around the hip joint, and the hip joint capsule is preserved intact. However, there is still a lack of high-quality evidence to support the superiority of the minimally invasive approach [9], so the choice between the traditional approach and the SP is highly controversial in terms of which one will provide better benefits to the adult patient.

Several relevant meta-analyses had analyzed the comparison of the efficacies of these two interventions before [10–13]. However, the conclusions of these meta-analyses were not identical and the outcome indicators included in these analyses were not comprehensive. Therefore, the objective of this study was to obtain more credible conclusions from a more detailed and comprehensive analysis of the two interventions by including a larger number of studies and patients. Therefore, this meta-analysis was a necessary update to confirm the effects of SP and could provide a more credible evidence-based reference for clinical practitioners.

## Methods

### Registration and protocol

This systematic review and meta-analysis followed the Preferred Reporting Items for Systematic Reviews and Meta-Analyses (PRISMA). The Meta-analysis has been registered on the PROSPERO platform (https://www.crd.york.ac.uk/PROSPERO/) with the registration number is CRD42022370701.

### Data source

We systematically searched PubMed, Embase, Cochrane, Web of Science, Chinese National Knowledge Infrastructure (CNKI), Wanfang Database and the reference list grey literature for studies published from the date of creation to December 2022. The computer-based search was based on a search formula using subject terms plus free words in each database, with the keyword and related Medical Subject Heading (MeSH) was used. The search keywords included "PA", "PLA", "posterior approach", "posterior lateral approach", "conventional approach" "supercapsular percutaneously assisted total hip" and "SuperPATH approach". Search strategies were detailed in Supplemental material 1.

### Selection criteria and study design

Inclusion criteria were as follows: patients received total hip arthroplasty through SP or PA due to failure of conservative treatment for hip-related disease; patients of any gender. Exclusion criteria were as follows: Patient’s age < 18 years; repeated publications; non-clinical trials; reviews, systematic reviews, meta-analyses, conference papers; the follow-up time is less than one month; no detailed description of the surgical approach; full text is not available; ongoing clinical trials not published in full. Only randomized controlled trials (RCTs) published in Chinese or English could be included.

### Outcome

There were 6 main research indicators: operation time (in minutes), incision length (in cm), intraoperative blood loss (in ml), length of hospital stay (in days), Harris hip score (HHS) and visual analogue score (VAS); There were 6 secondary research indicators: postoperative complications; time to start activities (in days), Barthel index (BI), 36-items short-form health survey scale (SF-36), postoperative acetabular cup angle (abduction angle and anteversion angle of the prosthesis (in degree)).

### Literature screening and data extraction

Based on the above inclusion and exclusion criteria, two researchers (Zhao, and Sun) screened the literature separately. Endnote (version 20) was applied to sort out the retrieved studies and eliminate the duplicate studies preliminarily, the titles and abstracts were read to exclude irrelevant studies, and then the full text was read to identify the initial included studies. Finally, the data were extracted independently from all eligible studies: basic information about the study including authors, year of publication, intervention, study type, outcome indicators, and characteristics of the populations (size and age). After completing these steps, two investigators' results were exchanged and reviewed with each other, and if any disagreement was encountered in the literature screening or data process, a third investigator will be arranged to participate in the discussion and consult on the inclusion of the article.

### Risk of bias

Two other researchers (Wang, and Xie) independently evaluate the Risk of bias of the included studies. In case of any disagreement, a third researcher will be assigned to participate in the discussion. The qualities of the included studies were assessed strictly according to the cochrane risk of bias assessment criteria (Cochrane RoB 2 tool) [14].

### Statistical methods

This meta-analysis was performed with Review Manage (version 5.4, Cochrane Collaboration) [15] and Stata (version 16.0, College Station, TX, USA: StataCorp LLC) [16]. The risk ratio (RR) and 95% Confidence Interval (95% CI) were used for dichotomous variables, and the weighted Mean Difference (MD) and 95% CI were used for continuous variables. The heterogeneity of different studies was tested by the P value of the Q-test and I^2^-test. If I^2^ < 50% and *P* > 0.05, the heterogeneity was suggested to be small, and a Fixed Effect model was used. If I^2^ > 50% or *P* < 0.05, the heterogeneity was suggested to be large, and the reasons for heterogeneity would be analyzed by sensitivity analysis. Sensitivity analysis was performed by the one-to-one study exclusion, and if the source of heterogeneity could not be identified, the random effect model was used. Publication bias tests were performed for those outcome indicators with included studies greater than 10, by making funnel plots and Begger's test, If *P* > *0.1*, the study was not considered to have publication bias, if *P* ≤ *0.1*, the study was considered to have publication bias.

## Result

### Study selection and characteristics

**Fig. 1:**
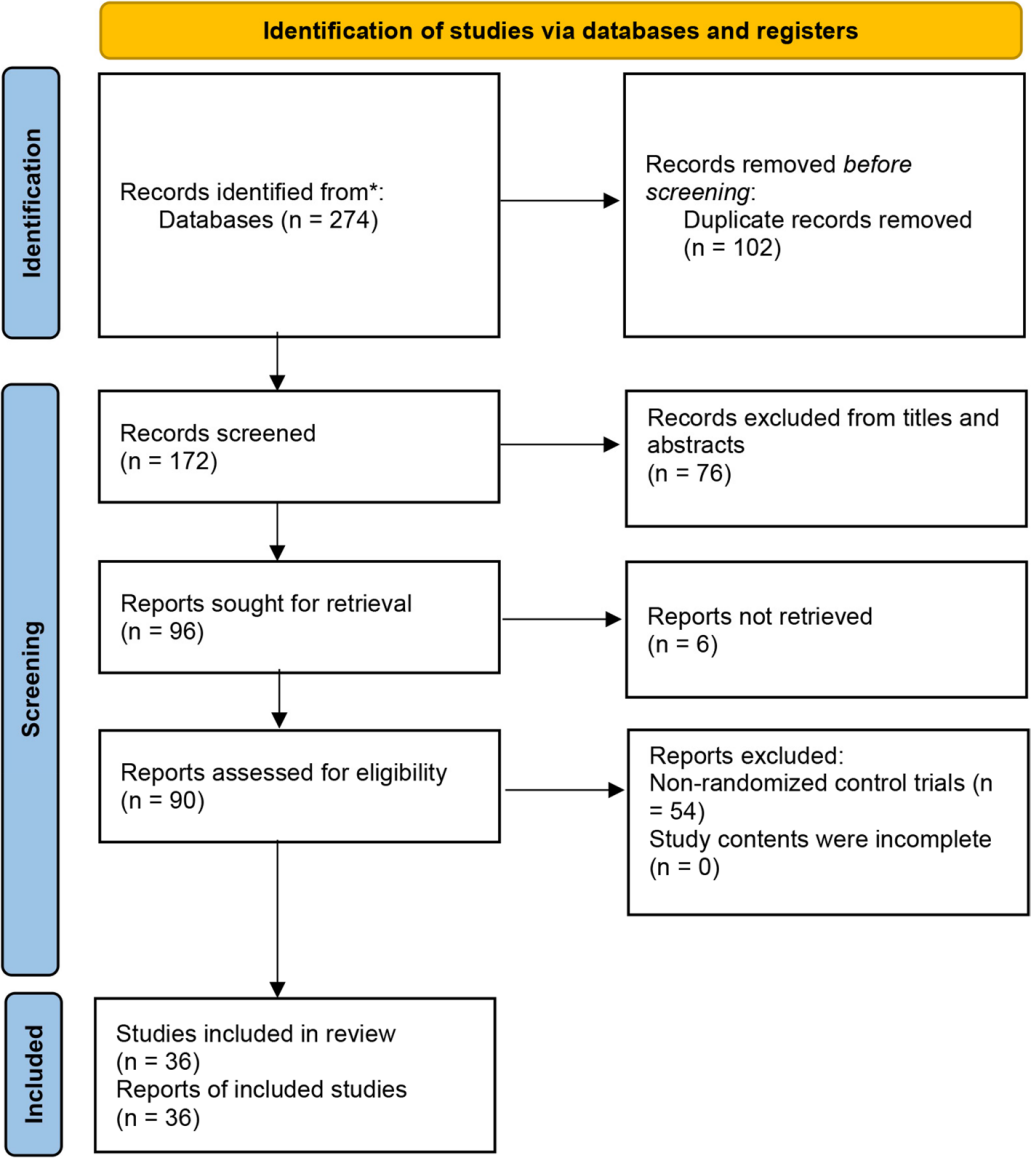
Flow diagram

According to the search strategy, a total of 274 studies were searched. 102 duplicate studies were excluded; 76 studies were excluded by reading the titles and abstracts; 96 relevant studies were assessed by reading the full text. 54 non-randomized controlled trials and 6 full texts could not be downloaded, and 36 studies were finally included. 5 studies [17–21] published in English and 31 studies [22–52] published in Chinese. The studies' screening process and results were shown in Fig. 1, and the basic characteristics of the included studies were shown in Supplementary Table 1.

### Risk of bias

**Fig. 2:**
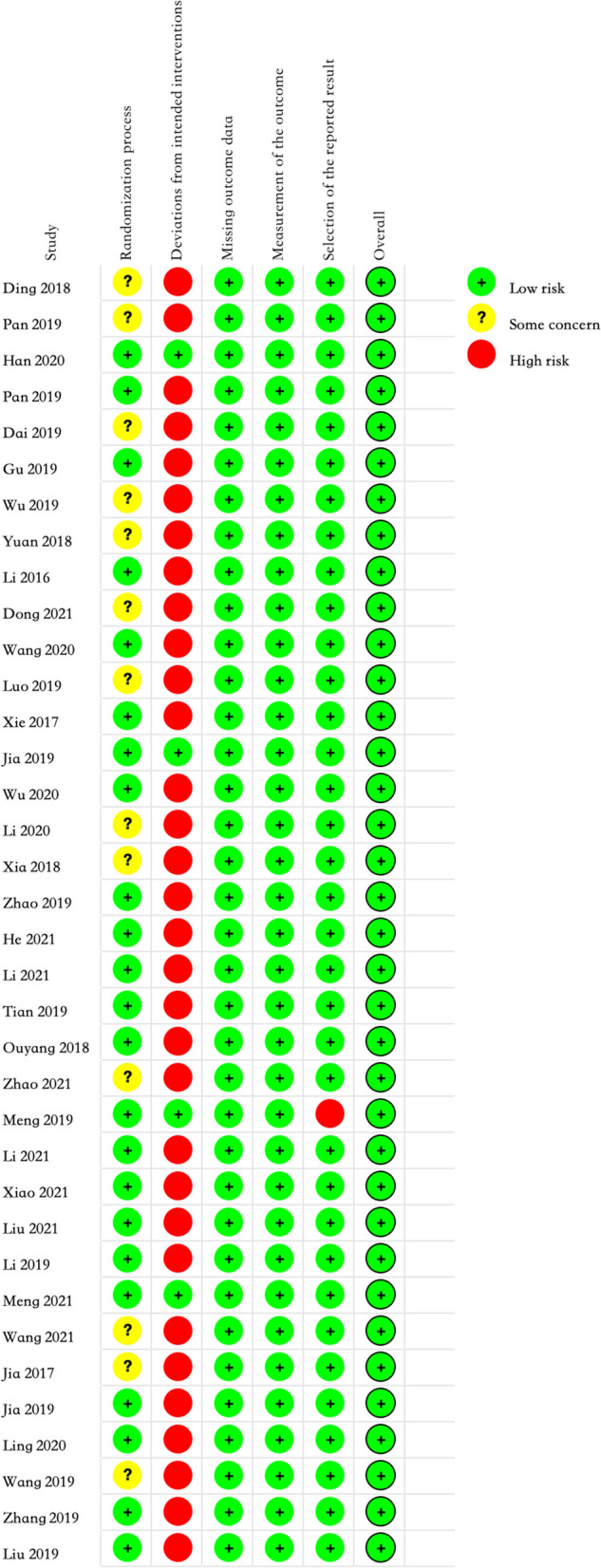
Risk of bias summary

**Fig. 3:**
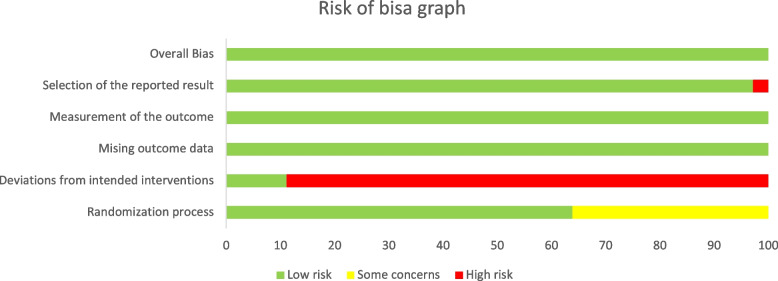
Risk of bias graph

The quality of the included studies was assessed according to the cochrane risk of bias assessment criteria (Cochrane RoB 2 tool) and the results are shown in Figs. 2 and 3.

### Main Outcome indicators

#### Operation time

**Fig. 4:**
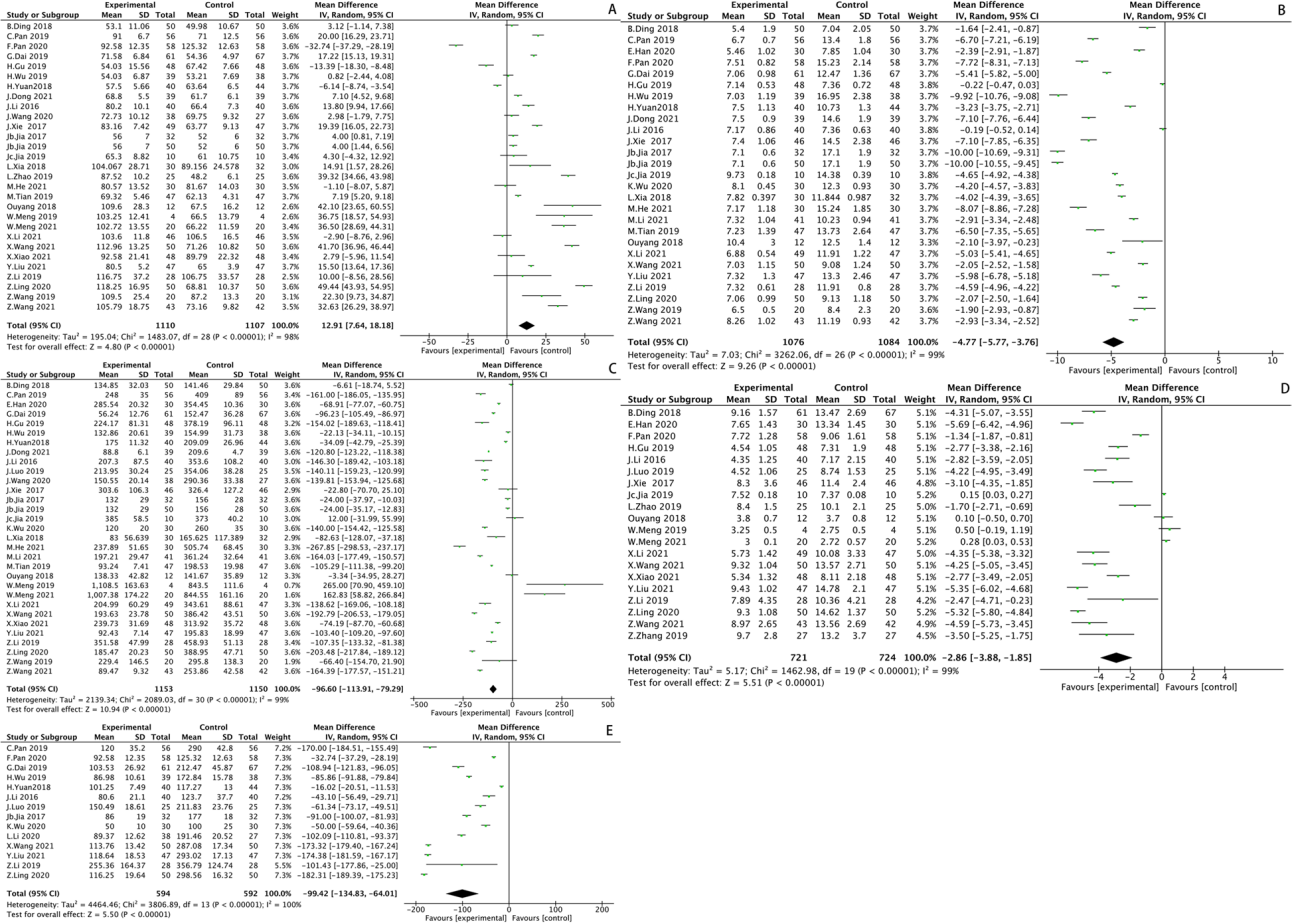
Operation time (**A**); Incision length (**B**); Intraoperative blood loss (**C**); The length of hospital stay (**D**); Time to start activities after surgery (**E**)

Operation time included 29 studies, and the result of the heterogeneity test was *I*^*2*^ = *98%, P* < *0.00001*, suggesting that there existed heterogeneity among the included studies, so sensitivity analysis should be conducted. No significant data deviation and no source of heterogeneity was found after analysis, suggesting that the results were relatively stable with low sensitivity, so a random-effects model was used for analysis. The results showed that the operation time of SP was 12.91 min longer than that of PA (*MD* = *12.91 [95%CI 7.64, 18.18], P* < *0.00001*). The result was shown in Fig. 4.

#### Incision length

Incision length included 27 studies, and the result of the heterogeneity test was *I*^*2*^ = *99%, P* < *0.00001*, suggesting that there existed heterogeneity among the included studies, so sensitivity analysis should be conducted. No significant data deviation and no source of heterogeneity was found after analysis, suggesting that the results were relatively stable with low sensitivity, so a random-effects model was used for analysis. The results showed that the incision length of SP was 4.77 cm shorter than that of PA (*MD* = *-4.77 [95%CI -5.77, -3.76], P* < *0.00001*). The result was shown in Fig. 4.

#### Intraoperative blood loss

Intraoperative blood loss included 31 studies, and the result of the heterogeneity test was *I*^*2*^ = *99%, P* < *0.00001*, suggesting that there existed heterogeneity among the included studies, so sensitivity analysis should be conducted. No significant data deviation and no source of heterogeneity was found after analysis, suggesting that the results were relatively stable with low sensitivity, so a random-effects model was used for analysis. The results showed that the volume of intraoperative blood loss of SP was 96.6 ml less than that of PA (*MD* = *-96.60 [95%CI 113.91, -79.29], P* < *0.00001*). The result was shown in Fig. 4.

#### The length of hospital stay

The length of hospital stay included 20 studies, and the result of the heterogeneity test was *I*^*2*^ = *99%, P* < *0.00001*, suggesting that there existed heterogeneity among the included studies, so sensitivity analysis should be conducted. No significant data deviation and no source of heterogeneity was found after analysis, suggesting that the results were relatively stable with low sensitivity, so a random-effects model was used for analysis. The results showed that the length of hospital stay of SP was 2.86 days shorter than that of PA (*MD* = *-2.86 [95% CI -3.88, -1.85], P* < *0.00001*). The result was shown in Fig. 4.

#### Harris Hip Score (HHS)

**Fig. 5:**
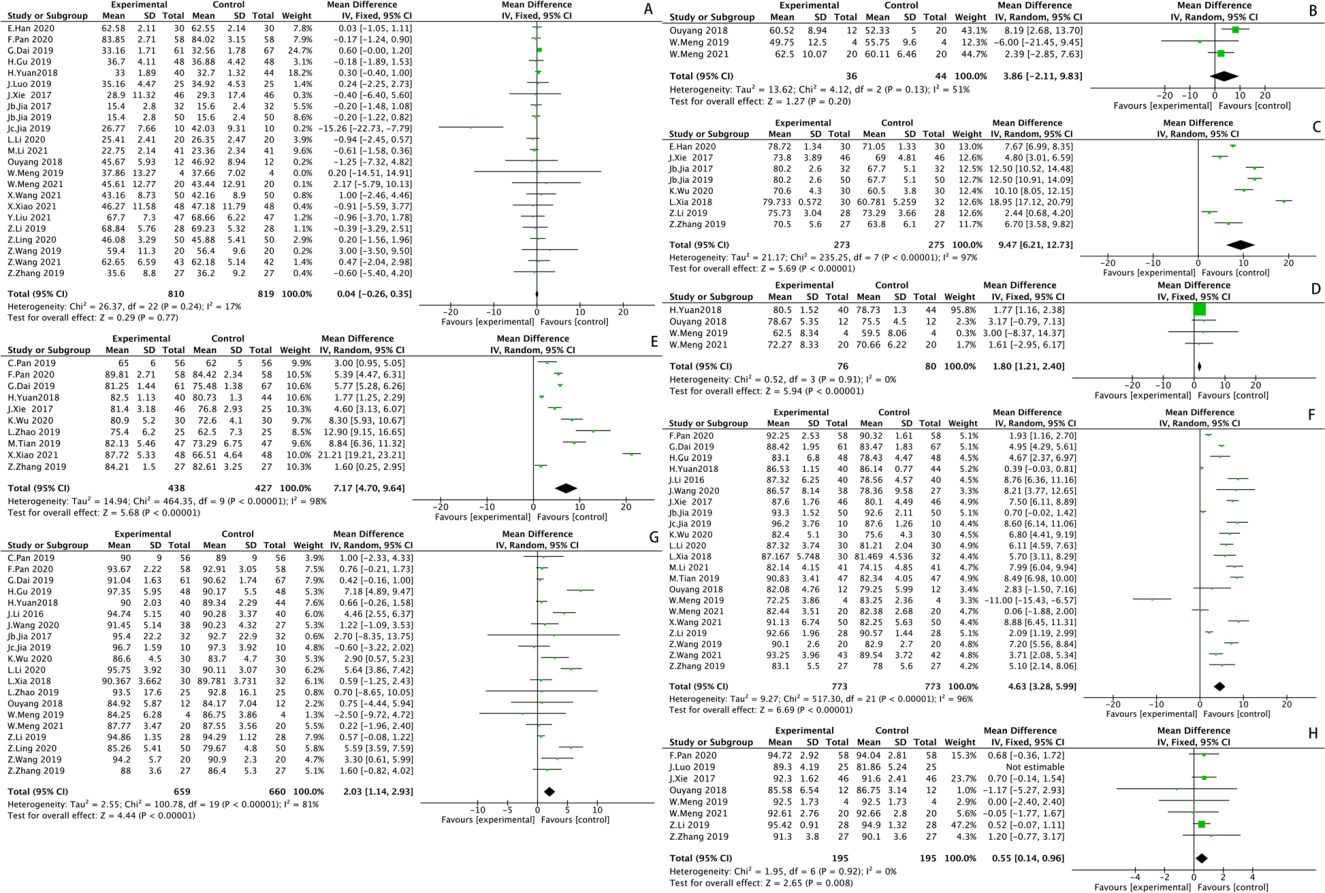
Harris Hip Score (HHS): Pre-operation (**A**); One day after surgery (**B**); One week after surgery (**C**); Two weeks after surgery (**D**); One month after surgery (**E**); Three months after surgery (**F**); Six months after surgery (**G**); One year after surgery (**H**)

Harris Hip Score (HHS) included nine subgroups at different time points, with 21 studies included preoperatively, 3 studies included one day postoperatively, 8 studies included one week postoperatively, 4 studies included two weeks postoperatively, 10 studies included one month postoperatively, 22 studies included three months postoperatively, 20 studies included six months postoperatively, and 8 studies included one year postoperatively. The results of the heterogeneity tests in each subgroup were: preoperatively (*I*^*2*^ = *17%, P* = *0.24*), one day postoperatively (*I*^*2*^ = *0%, P* = *0.91*), one week postoperatively (*I*^*2*^ = *97%, P* < *0.00001*), two weeks postoperatively (*I*^*2*^ = *89%, P* < *0.00001*), one month postoperatively (*I*^*2*^ = *98%, P* < *0.00001*), three months postoperatively (*I*^*2*^ = *96%, P* < *0.00001*), six months postoperatively (*I*^*2*^ = *81%, P* < *0.00001*), one year postoperatively *(I*^*2*^ = *75%, P* = *0.0003*). It was suggested that there was no heterogeneity between the studies included in the preoperative subgroup and one day postoperatively subgroup, so a fixed effect was used for the analysis. There existed heterogeneities in the remaining seven subgroups, so sensitivity analysis should be conducted. The analysis revealed that Luo2019 [44] had a greater effect on heterogeneity in one year postoperatively subgroup, and the results of the heterogeneity test performed again after excluding this study showed that there was no heterogeneity(*I*^*2*^ = *0%, P* = *0.92*), so a fixed-effect model was used. No studies with significant data bias or sources of heterogeneity were found in the remaining subgroups after analysis, suggesting relatively stable and less sensitive results, so a random-effect model was used for analysis. HHS of SP was higher than that of PA. One day postoperatively (*MD* = *3.86 [95%CI -2.11, 9.832], P* = *0.2*); three days postoperatively (*MD* = *6.79 [95%CI 1.41, 12.16], P* = *0.01*); one week postoperatively (*MD* = *9.47 [95%CI 6.21. 12.73], P* < *0.00001*); two weeks postoperatively (*MD* = *1.80 [95%CI 1.21, 2.40], P* < *0.00001*); one month postoperatively (*MD* = *7.17 [95%CI 4.70, 9.64], P* < *0.00001*); three months postoperatively (*MD* = *4.63 [95%CI 3.28, 5.99], P* < *0.00001*); six months postoperatively (*MD* = *2.03 [95%CI 1.14, 2.93], P* < *0.00001*); one year postoperatively (*MD* = *0.55 [95%CI 0.14, 0.96], P* = *0.008*). The results of the analysis were shown in Supplementary Table 2 and Fig. 5.

#### Visual Analogue Score (VAS)

**Fig. 6:**
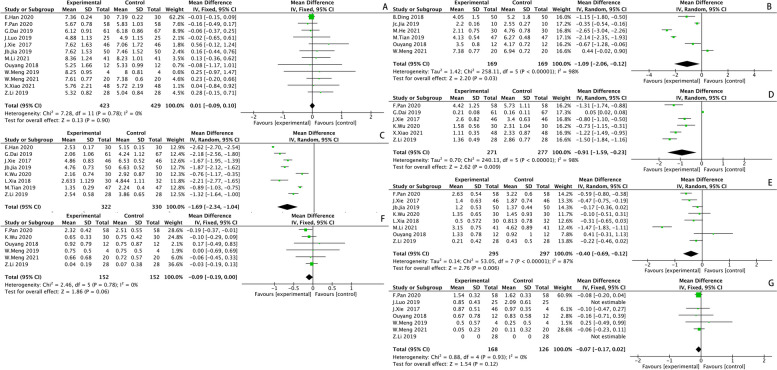
Visual Analogue Score (VAS): Pre-operation (**A**); One day after surgery (**B**); One week after surgery (**C**); One month after surgery (**D**); Three months after surger (**E**)y; Six months after surgery (**F**); One year after surgery (**G**)

Visual Analogue Score included seven subgroups at different time points, with 12 studies included preoperatively, 6 studies included one day postoperatively, 8 studies included one week postoperatively, 8 studies included one month postoperatively, 6 studies included three months postoperatively, 6 studies included six months postoperatively, and 7 studies included one year postoperatively. The results of the heterogeneity tests in each subgroup were: preoperatively (*I*^*2*^ = *0%, P* = *0.78*), one day postoperatively (*I*^*2*^ = *98%, P* < *0.00001*), one week postoperatively (*I*^*2*^ = *99%, P* < *0.00001*), one month postoperatively (*I*^*2*^ = *98%, P* < *0.00001*), three months postoperatively (*I*^*2*^ = *87%, P* < *0.00001*), six months postoperatively (*I*^*2*^ = *0%, P* = *0.78*), one year postoperatively *(I*^*2*^ = *91%, P* = *0.0003*). It was suggested that there was no heterogeneity between the studies included in the preoperative subgroup and six months postoperatively subgroup, so a fixed effect was used for the analysis. There existed heterogeneities in the remaining five subgroups, so sensitivity analysis should be conducted. The analysis revealed that Luo2019 [44] had a greater effect on heterogeneity in one year postoperatively subgroup, and the results of the heterogeneity test performed again after excluding this study showed that there was no heterogeneity (*I*^*2*^ = *0%, P* = *0.93*), so a fixed-effect model was used. No studies with significant data bias or sources of heterogeneity were found in the remaining subgroups after analysis, suggesting relatively stable and less sensitive results, so a random-effect model was used for analysis. The VAS of SP was less than that of PA. One day postoperatively (*MD* = *-1.09 [95%CI -2.06, -0.12], P* = *0.03*); one week postoperatively (*MD* = *-1.69 [95%CI -2.34, -1.04], P* < *0.00001*); one month postoperatively (*MD* = *-0.91 [95%CI -1.59, -0.23], P* = *0.009*); three months postoperatively (*MD* = *-0.40 [95%CI -0.69, -0.12], P* = *0.006*); six months postoperatively (*MD* = *-0.09 [95%CI -0.19, 0.00], P* = *0.06*); one year postoperatively (*MD* = *-0.07 [95%CI -0.17, -0.02], P* = *0.12*). The results of the analysis were shown in Supplementary Table 2 and Fig. 6.

### Secondary outcome indicators

#### Postoperative complications

**Fig. 7:**
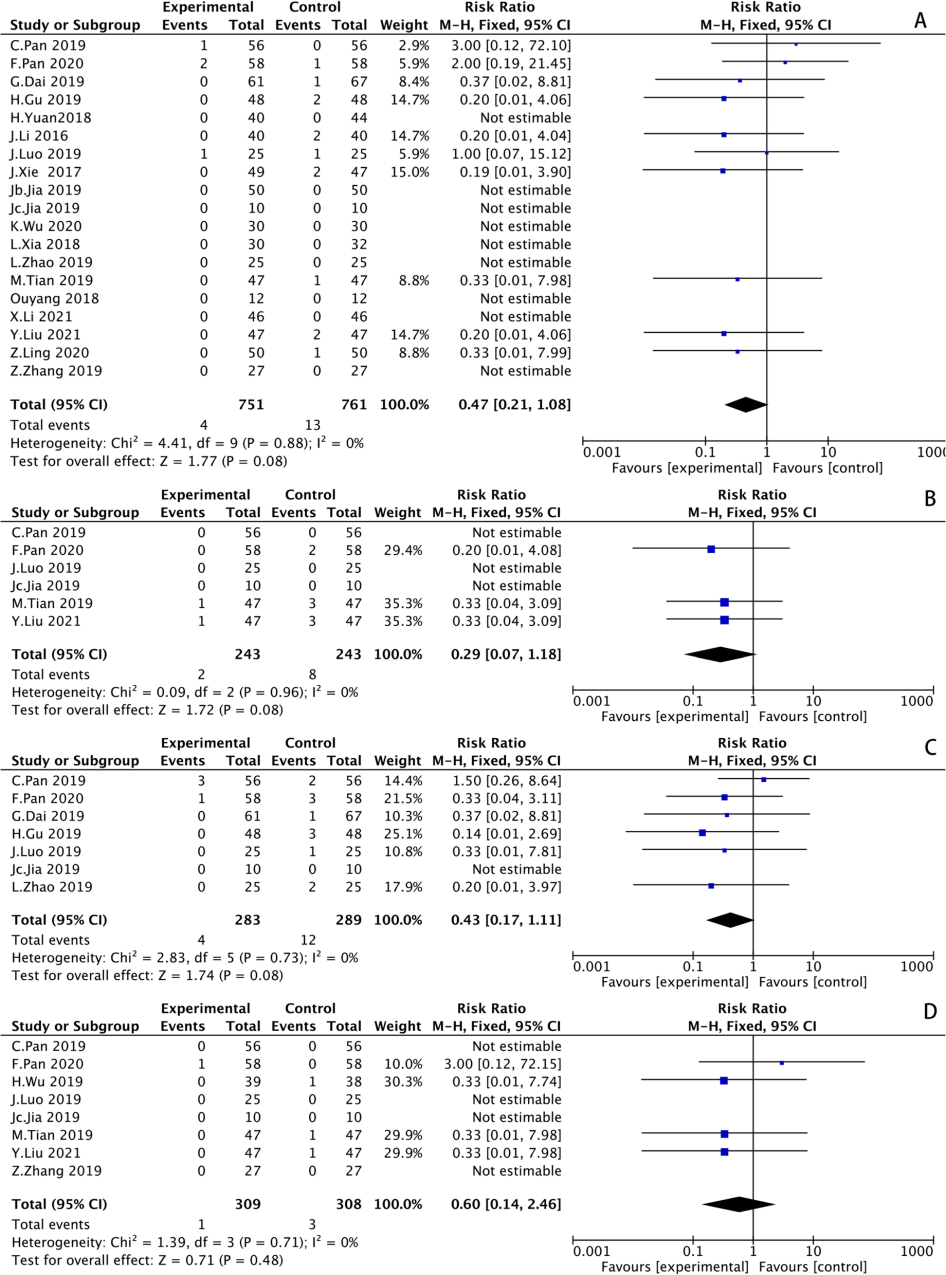
Postoperative complications. Prosthetic joint dislocation (**A**); Postoperative periprosthetic infection subgroup (**B**); Deep vein thrombosis of the lower limbs (**C**); Sciatic nerve injury (**D**)

Postoperative complications included 4 major studies, with 19 studies in the postoperative prosthetic joint dislocation subgroup, 7 studies in the postoperative deep vein thrombosis of the lower limbs subgroup, 8 studies in the postoperative sciatic nerve injury subgroup, and 6 studies in the postoperative periprosthetic infection subgroup. The results of the heterogeneity tests were:* I*^*2*^ = *0%, P* = *0.88; I*^*2*^ = *0%, P* = *0.73; I*^*2*^ = *0%, P* = *0.71* and *I*^*2*^ = *0%, P* = *0.96* respectively, suggesting that there was no heterogeneity among the studies included in these four subgroups. The results were shown in Supplementary Table 3 and Fig. 7.

#### Barthel Index (BI)

Barthel index included four subgroups at different time points, with 4 studies included preoperatively, 3 studies included one week postoperatively, 3 studies included three months postoperatively, and 3 studies included one year postoperatively. The results of the heterogeneity tests in each subgroup were: preoperatively (*I*^*2*^ = *76%, P* = *0.71*), one week postoperatively (*I*^*2*^ = *57%, P* = *0.01*), three months postoperatively (*I*^*2*^ = *78%, P* = *0.01*), one year postoperatively (*I*^*2*^ = *94%, P* < *0.00001*). The results suggested that there existed heterogeneities among the studies included in the four subgroups, but the number of studies included in each subgroup was limited, so sensitivity analyses were not performed, so a random-effect model was used for all analyses. BI of SP was higher than that of PA. One week postoperatively (*MD* = *6.44 [95%CI 2.75, 10.13], P* = *0.0006*); three months postoperatively (*MD* = *6.17 [95%CI 1.89, 10.44], P* = *0.005*); one year postoperatively (*MD* = *4.22 [95%CI—2.49, 10.93], P* = *0.005*). The results of the analysis were shown in Supplementary Fig. 1.

#### SF-36 score

SF-36 score included two subgroups, with 5 studies included preoperatively, and 7 studies included postoperatively. The results of the heterogeneity tests in each subgroup were: preoperatively (*I*^*2*^ = *0%, P* = *1.0*), and postoperatively (*I*^*2*^ = *98%, P* < *0.00001*). It was suggested that there was no heterogeneity between the studies included in the preoperative subgroup, so a fixed effect was used for the analysis. There existed heterogeneities among the studies included in the postoperative subgroup, but the number of studies included was limited, so sensitivity analyses were not performed, and a random-effect model was used for all analyses. SF-36 score of SP was higher than that of PA. Postoperatively (*MD* = *9.66 [95%CI 3.52, 15.80], P* = *0.002*); The results of the analysis were shown in Supplementary Fig. 1.

#### Time to start activities after surgery

Time to start activities after surgery included 12 studies, and the result of the heterogeneity test was I2 = 99%, *P* < 0.00001, suggesting that there existed heterogeneity among the included studies, so sensitivity analysis should be conducted. No significant data deviation and no source of heterogeneity was found after analysis, suggesting that the results were relatively stable with low sensitivity, so a random-effect model was used for analysis. The results showed that the time to start activities after surgery of SP was 2.34 days earlier than that of PA (MD = -2.34 [95%CI -2.96, -1.72], *P* < 0.00001). The result was shown in Fig. 4.

#### Postoperative acetabular cup angles

Postoperative acetabular cup angles included two subgroups, with 10 studies included in the abduction angle of the prosthesis subgroup and 6 studies included in the anteversion angle of the prosthesis subgroup. The results of the heterogeneity tests in each subgroup were: the abduction angle subgroup (*I*^*2*^ = *79%, P* < *0.00001*); the anteversion angle subgroup (*I*^*2*^ = *0%, P* = *0.87*). The results suggested that there was no heterogeneity between studies included in the anteversion angle subgroup, so a fixed effect was used for the analysis. There was heterogeneity between studies included in the abduction angle subgroup. No studies with significant data bias and sources of heterogeneity were identified after analyzing, suggesting relatively stable and low sensitivity results, so a random-effect model was used for analysis. Adduction angle (*MD* = *-0.26 [95%CI -1.87, 1.34], P* = *0.75*); anteversion angle (*MD* = *-0.81 [95%CI -1.30, -0.33], P* = *0.001*). The results of the analysis were shown and Supplementary Fig. 2.

#### Publication bias

Publication bias was assessed by making funnel plots and Egger's tests for the outcome indexes including more than 10 studies. The funnel plots of postoperative dislocation of the prosthetic joint (*p* = *0.105*), operative time (*p* = *0.542*), intraoperative blood loss (*p* = *0.356*), the anteversion angle (*p* = *0.289*), and one months postoperatively HHS (*p* = *0.190*) were relatively symmetrical and there existed no publication bias. Publication bias existed in the length of hospital stay(*p* = *0*), incision length (*p* = *0.011*), six months postoperatively HHS (*p* = *0.07*), and three months postoperatively HHS (*p* = *0.007*). The results of the sensitivity analysis showed that most of the indicators had no publication bias and a few indicators had publication bias. The results were shown in Supplementary Fig. 3.

## Discussion

This study included 36 qualified RCT studies. In this meta-analysis, we analyzed the results of total hip arthroplasty performed through the SuperPATH and conventional posterior lateral/ posterior approaches in adult patients. It improved our understanding of the use of the SuperPATH. We found that the main advantage of the SP was that this approach reduced surrounding tissue destruction, resulting in less surgical trauma to the patient and a faster return to function. We newly found that there was a significant improvement in postoperative hip function (HHS) and functional status in activities of daily living (BI) within three months postoperatively, and a significant reduction in the level of hip pain (VAS) within one month postoperatively. Patients also showed a significant improvement in mental health and overall health (SF-36) postoperatively. There was no difference in the occurrence of postoperative complications between the two methods. The differences in hip function (HHS) after postoperative six months and hip pain level (VAS) after postoperative three months were very small. However, the PA took less time to operate and had a higher accuracy of prosthesis placement.

In a meta-analysis [10] published by Ramadanov et al. in 2020, it was shown that no differences in terms of postoperative acetabular cup angle, intraoperative blood loss, length of hospital stay, and postoperative complications. In a meta-analysis [13] published by Y.Ge et al. in 2021, the analysis showed no difference in acetabular abduction angle between the two groups. In a meta-analysis [12] published by V.M. Joseph et al. in 2022, it was concluded that no significant improvement in VAS or HHS. In a meta-analysis [11] published by Ramadanov et al. in 2022, it was concluded that intraoperative blood loss was lower and HHS was higher in SuperPATH. There were no differences in postoperative complications between the two methods, with lower VAS and shorter incision length. The conclusions of these meta-analyses were not identical and the outcome indicators included in these analyses were not comprehensive, so the previous meta-analyses were hardly convincing. Therefore, this meta-analysis was a necessary update to confirm the effects of SP. This meta-analysis has many advantages over previous systematic studies. The inclusion of the largest number of RCT studies in this study significantly increased the sample size of the analysis, thus allowing for a more comprehensive comparison between the SP approach and the PA approach. A total of six primary and six secondary indicators were included, covering a more comprehensive set of outcome indicators reflecting secondary surgical trauma, speed of recovery of postoperative hip function, surgical efficacy, incidence of postoperative complications, accuracy of prosthesis implantation, functional status of the patient, and postoperative overall health. We added multiple time points to the analyses of HHS and VAS to produce more detailed results. More importantly, we performed detailed analyses of metrics that were not analysed in previous meta-analyses due to study size limitations but were clinically significant. We hoped to provide a more credible evidence-based reference for clinical practitioners.

In terms of the operation time, the SP was 12.91 min longer than the PA. By analyzing the outcome indexes of different studies, we found that the time of different operators to complete the operation via the SP was significantly different. The longest operation time was 118.25 min [53] and the shortest was 53.1 min [22]. Therefore, we believe that the operative time of the SP approach may correlate with the operator's mastery, which explained the large heterogeneity between the included researches for this outcome indicator. GS. Qiao et al. reported that the learning time of the SP had a learning curve pattern [54]. Before the operator operated 30 cases of SP surgery, the operative time of the SP was longer than that of PA; after operating 30 cases of SP surgery, the operating time would decline continuously; after operating 60 cases of surgeries, the operating time of the SP would be less than that of the PA. In the study reported by K.J. Rasuli and W. Gofton, it was found that the operation time of SuperPATH became shorter as the number of operations increased and continued to decrease. Even when the number of operations exceeded 50, the operation time continued to decrease [55]. It suggested that SP was still a relatively new method for many physicians at this stage, but it was highly learnable, and the operation time for SP would be reduced after the surgeon gained enough surgical experience.

The incision length of the SP was 4.77 cm shorter than that of the PA. Only a smaller incision was required because the operators polished the acetabulum and placed the prosthesis through a trocar and connecting rod. This indicator was highly heterogeneous, probably because incision length was influenced by patients’ height, weight, age, and gender, so there was a large variability among the included studies. It was commonly believed that limited intraoperative field exposure would lead to an increased incidence of postoperative prosthetic dislocation, and the prolonged operative time would lead to longer exposure to the surgical site, which could be more prone to infection [56]. However, according to the analysis of postoperative complications, there was no difference in the occurrence of postoperative complications such as prosthesis dislocation and periprosthetic infection between the two methods, indicating that the SuperPATH did not lead to an increased incidence of postoperative complications. We supposed that the reason was there was no need to sever any muscle during surgery and the joint capsule was preserved intact, which reduced surgical trauma and thus reduced the chance of intraoperative infection. No intraoperative dislocation of the hip joint was needed, thus the physiological anatomy was better preserved so the incidence of prosthesis dislocation was lower [57].

A common indicator of the accuracy of the implant position was the postoperative acetabular cup angle (abduction angle, anteversion angle). G.E.Lewinnek et al. indicated that the lowest rate of prosthesis dislocation occurred when the angle was 5° to 25° of anteversion and 30° to 50° of abduction [58]. The surgical incision of the SP was much smaller than that of the PA. A smaller surgical incision meant a smaller field of view for manipulation, and the SP without severing any of the muscle also increased the difficulty of implanting the prosthesis and increased the rate of misalignment of the prosthesis. By analyzing the results, the SP did not increase the misalignment rate. The anteversion angle was 0.81° greater in the PA than in the SP, and there was no difference in the abduction angle between the two methods (*P* > 0.05). However, by looking at the results of each included study, it was found that the SP was slightly less accurate in implanting the prosthesis than the PA, but the prosthesis was still within the ideal angulation range. The angulation ranges of the implant were within the ideal range for both methods, but the SP approach was slightly less accurate than the PA and did not show superiority. This was the same finding as a study reported by Filippo Migliorini et al. for a minimally invasive approach to total hip arthroplasty did not show superior results in postoperative radiographic findings compared to conventional approaches [59]. This was because of the need to use some new instruments and the small intraoperative field exposure, which could increase the difficulty of implanting the prosthesis. The use of the transversal acetabular ligament to guide the joint cup alignment and individualized positioning of the acetabular cup during surgery may reduce this effect [60].

The intraoperative blood loss of the SP was 96.60 ml less than that of the PA, indicating that the SP could lead to less blood loss in patients. The reason for this was that the SP mostly used blunt separation of the distraction muscle groups and did not require any muscles to be destroyed to expose the joint capsule. In contrast, the PA required the dissection of multiple muscle groups to expose the joint capsule. Notably, K. Xu et al. reported that SP produces more invisible blood loss [61]. The possible reasons they analyzed were the intraoperative destruction of bone trabeculae that aggravated the intramedullary hemorrhage and the incomplete hemostasis due to the small surgical field. Therefore, surgeons needed to be concerned about possible hidden blood loss when performing surgery through the SP. Intraoperative blood loss was highly heterogeneous which may be influenced by different surgeons, patients’ BMI, and underlying disease.

The results showed a reduction in hospital stay of 2.86 days in the SP and 2.34 days earlier start of the postoperative activity compared with PA. The SP approach was a minimally invasive approach that produced less surgical trauma and lower tissue edema due to local inflammatory response than PA. This speeded up the recovery of their ability to perform daily activities and allowed for a shorter hospital stay. Length of hospital stay was highly heterogeneous because the length of stay was affected by hospital protocols, but all included studies demonstrated a shorter length of hospital stay for SP than PLA. A study reported by Chow et. al. showed the same advantage of SP that the total hospital costs were lower in the SP group than in the PA, with an average reduction in surgical costs in the SP group of 15.0%, the average length of stay was reduced by 1.4 days, and readmission rate reduced by 2.5% [62].

The Harris Hip Score(HHS) was a specific index used to evaluate the hip function, ranging from 1 to 100, with higher scores representing better hip function [63]. The results of the analysis showed that there was no difference between the two methods preoperatively (*p* > 0.05). The HHS was higher in the SP compared to the PA at 3.86 one day postoperatively, 6.79 three days postoperatively; 9.47 one week postoperatively; 7.17 one month postoperatively; 4.63 three months postoperatively; 2.03 six months postoperatively; and 0.55 one year postoperatively. We observed a substantial increase in HHS in the SP within three months postoperatively. After postoperative six months, the HHS difference decreased to nearly 2 points. This suggested that the main advantage of the SP was that it allowed for a more rapid recovery of postoperative hip function and an early return to normal life for the patients, which was more significant in the elderly population.

The Visual Analogue Score(VAS) score was used to measure the degree of pain, ranging from 0–10, with lower scores representing less pain [64]. The analysis showed that there was no difference between the two methods preoperatively (*p* > 0.05). VAS was lower in the SP group compared to the PA at 1.09 one day postoperatively; 1.69 one week postoperatively; 0.91 one month postoperatively; 0.40 three months postoperatively; 0.09 six months postoperatively; and 0.07 one year postoperatively. We observed that there was a substantial reduction in the VAS of the SP within one month postoperatively. The difference in HHS reduced gradually to less than 0.5 points after postoperative three months. This was associated with less secondary surgical trauma and soft tissue injuries in the SP. The advantage of the SP technique was that it allowed patients to significantly reduce hip pain early. The reduction in pain could reduce the use of pain medication, anxiety, bedtime, and complications such as decubitus ulcers and deep vein thrombosis of the lower limbs, which were meaningful to patients.

The Barthel Index(BI) was an indicator used to evaluate the patient's ability to perform activities of daily living [65]. The SF-36 scale was used for the patient's evaluation of self-health, mental health, and general well-being [66]. These two indicators reflected patients' subjective assessment of the degree of improvement in hip function and their satisfaction with the surgery. Results showed that the Barthel Index was higher in the SP than in the PA, and was more significant within three months postoperatively. The SF-36 scores were significantly higher in the SP than in the PA. It showed the higher satisfaction of patients in the SP with their postoperative daily living.

We found no publication bias in most of the outcomes, but publication bias existed in the length of hospital sty(*p* = 0), incision length (*p* = 0.011), six months postoperatively HHS (*p* = 0.07), and three months postoperatively HHS (*p* = 0.007). There was a strong publication bias in the length of hospital stay, we supposed that the reason may be that a new operative approach was usually firstly introduced to high-level hospitals. However, in high-level hospitals, the actual length of hospital stays was influenced not only by the patients' status of postoperative recovery but also by hospital protocols to some extent. In China, the Diagnosis Related Groups (DRGs) protocol required a faster bed turnover in higher-level hospitals, which may lead to a short length of hospital stay. By observing the results reported, most studies reported a shorter length of hospital stay for SP, so we supposed that the analysis result was plausible and the effect of publication bias was small. There existed a strong publication bias in the outcomes of the time to start activity postoperatively and incision length, both of which could be influenced by differences in bone cement, differences in hip implants, and differences in the patient's underlying diseases, age, gender, and BMI to some extent [67]. For example, women had longer incision lengths than men; heavier patients had longer incision lengths than those with less weight; patients with more underlying diseases required a longer time to get out of bed. The RCT studies included in this study failed to achieve uniformity in the above-influencing factors, and the result of Egger's test of the preoperative HHS was 0.034, suggesting a variation in the status of patients preoperatively. By observing the results of the time to start activity postoperatively and incision length reported by studies included, all studies reported a shorter incision length for SP and an earlier time to start activities for SP than for PA, suggesting the analysis was plausible and the publication bias had less impact on the results. There was some publication bias in the HHS three months postoperatively, we supposed that the reason may be that the Harris Hip Scale was scored by researchers, so scores were influenced by the subjectivity of the evaluators, which led to the variation between the results reported in different studies. By observing the results reported, almost all studies reported a higher score of HHS for SP than PA, suggesting the analysis was plausible and the impact of publication bias was small. Furthermore, SP was still a relatively new method for most clinicians, and the number of RCTs reported was still small, so the sample sizes included were not yet large enough, which may have contributed to publication bias and may have had an impact on the reliability of the results analyzed. We will continue to review the subsequent publications of relevant RCTs, and the subsequent publications of more high-quality relevant RCTs will reduce the publication bias in these results.

There were some limitations to this meta-analysis: (1) There existed heterogeneities and publication bias in a few of the outcomes which may influence the analyses. (2) Fewer outcome indicators included smaller sample sizes, and these may have some impact on the results. (3) A larger proportion of studies published in Chinese were included in this meta-analysis, which may have had some impact on the results of the study.

## Conclusion

Compared to PA, the SuperPATH technique results in smaller surgical incisions, less intraoperative blood loss, shorter hospital stays, and less surgical trauma. Its greatest advantage is accelerated recovery of hip function and improved overall quality of life after surgery. However, it requires a longer operative time and the accuracy of the prosthesis implantation is not as good as that of the PA. Therefore, the SuperPATH requires continuous learning by the surgeon in order to minimise the impact of its shortcomings.

### Supplementary Information

**Additional file 1: Supplementary material 1.** Search Strategy.

**Additional file 2:** **Supplementary Table 1.** **Supplementary Table 2.** **Supplementary Table 3.**

**Additional file 3:** **Supplementary Figure 1.** **Supplementary Figure 2.** **Supplementary Figure 3.** 

## Data Availability

All data generated or analysed during this study are included in this published article and its supplementary information files and all data and materials in this article were available.
