# Supplementary material for: Comparison of clinical outcomes of supercapsular percutaneously-assisted approach total hip arthroplasty versus conventional posterior approach for total hip arthroplasty in adults: a systematic review and meta-analysis
Source: BMC Musculoskelet Disord. 2024 Jan 2;25:25. doi: 10.1186/s12891-023-07126-x (PMC10759432; doi:10.1186/s12891-023-07126-x)
Supplement: Supplementary file 1 — Additional file 1: Supplementary material 1. Search Strategy. [file 12891_2023_7126_MOESM1_ESM.docx]

| **Datebase** | **Search strategy** |
| --- | --- |
| **Pubmed** | ((posterior lateral approach [Title/Abstract]) OR (PLA [Title/Abstract]) OR (posterolateral total hip arthroplasty [Title/Abstract]) OR (posterior approach [Title/Abstract]) OR (PA [Title/Abstract])) AND ((superpath [Title/Abstract]) OR (supercapsular percutaneously assisted total hip [Title/Abstract])) |
| **Web of science** | (TS = (((posterior lateral approach) OR (PLA) OR (posterolateral total hip arthroplasty) OR (posterior approach) OR (PA)))) AND TS = (((superpath approach) OR (supercapsular percutaneously assisted total hip))) |
| **Embase** | #1 'posterior lateral approach': ab,ti OR 'pla': ab,ti OR 'posterior approach': ab,ti OR 'pa': ab,ti  #2 'superpath approach': ab,ti OR 'supercapsular percutaneously assisted total hip': ab,ti  #3 #1 AND #2 |
| **Cochrane** | #1 (posterior approach):ti,ab,kw OR (PA):ti,ab,kw OR (posterior lateral approach):ti,ab,kw OR (PLA):ti,ab,kw (Word variations have been searched)  #2 (supercapsular percutaneously assisted total hip):ti,ab,kw OR (SuperPATH approach):ti,ab,kw (Word variations have been searched)  #3 #1 AND #2 |
| **CNKI** | （主题：后入路（精确））OR（主题：后外侧入路（精确））OR（主题：PA（精确））OR（主题：PLA（精确）） AND （（主题：superpath入路（精确））OR（主题：关节囊外经皮辅助全髋关节置换术（精确））） |
| **Wanfang Database** | [主题: (后入路 OR 后外侧入路 OR PA OR PLA) AND 主题: (superpat入路 OR 关节囊外经皮辅助全髋关节置换术)](http://wfo.lib.ustc.edu.cn/librarian/*) |

Supplementary material1. Search Strategy
