# Supplementary material for: Comparison of clinical outcomes of supercapsular percutaneously-assisted approach total hip arthroplasty versus conventional posterior approach for total hip arthroplasty in adults: a systematic review and meta-analysis
Source: BMC Musculoskelet Disord. 2024 Jan 2;25:25. doi: 10.1186/s12891-023-07126-x (PMC10759432; doi:10.1186/s12891-023-07126-x)
Supplement: Supplementary file 2 — Additional file 2: Supplementary Table 1. Supplementary Table 2. Supplementary Table 3. [file 12891_2023_7126_MOESM2_ESM.docx]

| 1st Author and Year | Study design | Sample size | | Gender (M/F) | | Mean age (SD/range)（M/F） | | Approach | |
| --- | --- | --- | --- | --- | --- | --- | --- | --- | --- |
|  |  | EG | CG | EG | CG | EG | CG | EG | CG |
| Jb. Jia 2019 | RCT | 50 | 50 | 31/19 | 29/21 | 78.10±2.30 | 79.5±2.6 | SP | PA |
| 1. Li   2020 | RCT | 30 | 30 | 16/14 | 18/12 | 70.35+4.26 | 70.12+4.78 | SP | PA |
| X. Li  2021 | RCT | 49 | 47 | 27/22 | 24/23 | 75.53±7.34 | 77.21±7.84 | SP | PA |
| 1. Meng 2021 | RCT | 20 | 20 | 8/12 | 9/11 | 64.55±9.06 | 65.25±10.33 | SP | PA |
| 1. Meng   2019 | RCT | 4 | 4 | 4/0 | 4/0 | 51±4.54 | 51±4.54 | SP | PA |
| 1. Xie 2017 | RCT | 46 | 46 | 34/12 | 27/19 | 66.60±11.88 | 64.47±12.09 | SP | PA |
| 1. Ding 2018 | RCT | 50 | 50 | 22/28 | 21/29 | 81.18±5.93 | 80.76±5.57 | SP | PA |
| D. Dai  2019 | RCT | 61 | 67 | 22/39 | 25/43 | 65～80 | 65～79 | SP | PA |
| 1. He   2021 | RCT | 30 | 30 | 18/12 | 16/14 | 42.10±5.60 | 43.06±5.41 | SP | PA |
| 1. Ling   2020 | RCT | 50 | 50 | 31/19 | 29/21 | 89.14±3.60 | 88.95±3.71 | SP | PA |
| Y. Liu  2021 | RCT | 47 | 47 | 26/21 | 24/23 | 68.27±3.71 | 68.55±3.40 | SP | PA |
| 1. Wu   2020 | RCT | 30 | 30 | 20/10 | 15/15 | 71.2±3.1 | 71.2±3.2 | SP | PA |
| 1. Wu   2019 | RCT | 39 | 38 | 22/17 | 23/15 | 69.51±3.78 | 70.19±3.13 | SP | PA |
| 1. Han | RCT | 30 | 30 | 16/14 | 15/15 | 68.52±1.42 | 68.51±1.44 | SP | PA |
| 1. Xia   2018 | RCT | 30 | 32 | 8/22 | 11/21 | 81.00±4.57 | 80.66±4.26 | SP | PA |
| Z. Zhang  2019 | RCT | 27 | 27 | 10/17 | 12/15 | 62.41±6.44 | 61.28±6.70 | SP | PA |
| Z. Li  2019 | RCT | 28 | 28 | 15/13 | 16/12 | 66.64±9.56 | 65.35±7.77 | SP | PA |
| 1. Li   2021 | RCT | 41 | 41 | 19/22 | 18/23 | 75.3±4.35 | 76.46±5.14 | SP | PA |
| J. Li  2016 | RCT | 40 | 40 | 27/13 | 24/16 | 67.3±7.05 | 70.2±6.25 | SP | PA |
| OuYang  2018 | RCT | 12 | 12 | 8/4 | 9/3 | 45～71 | 47～67 | SP | PA |
| 1. Pan   2019 | RCT | 56 | 56 | 29/27 | 26/30 | 68±4 | 65±3 | SP | PA |
| 1. Pan   2020 | RCT | 58 | 58 | 34/24 | 33/25 | 62.31±6.84 | 65.62±6.96 | SP | PA |
| X. Wang  2021 | RCT | 50 | 50 | 26/24 | 27/23 | 67.84±7.3 | 67.15±6.58 | SP | PA |
| Z. Wang  2019 | RCT | 20 | 20 | 12/8 | 11/9 | 47.1±15.8 | 46.9±16.2 | SP | PA |
| M. Tian  2019 | RCT | 47 | 47 | 29/18 | 30/17 | 62.31±8.39 | 61.85±7.13 | SP | PA |
| J. Luo  2019 | RCT | 25 | 25 | 16/9 | 15/10 | 54.37±8.81 | 54.96±8.73 | SP | PA |
| H. Yuan  2018 | RCT | 40 | 44 | 24/16 | 21/23 | 67～79 | 69～82 | SP | PA |
| Jc. Jia  2019 | RCT | 10 | 10 | 2/8 | 5/5 | 70.0±0.32 | 70.8±2.28 | SP | PA |
| 1. Zhao   2019 | RCT | 25 | 25 | 12/13 | 10/15 | 81.5±5.2 | 82.8±6.3 | SP | PA |
| H. Gu  2019 | RCT | 48 | 48 | 32/16 | 29/19 | 63.06±13.58 | 65.81±13.75 | SP | PA |
| J. Wang  2020 | RCT | 38 | 27 | 16/22 | 11/16 | 76.54±5.32 | 75.24±4.32 | SP | PA |
| Z. Wang  2021 | RCT | 43 | 42 | 26/17 | 24/18 | 71.53±3.76 | 71.58±3.79 | SP | PA |
| X. Xiao  2021 | RCT | 48 | 48 | 28/20 | 27/21 | 72.79±3.51 | 73.42±3.45 | SP | PA |
| J. Dong  2021 | RCT | 39 | 39 | 18/21 | 19/20 | 70.4±6.9 | 72.0±6.7 | SP | PA |
| Jb. Jia  2017 | RCT | 32 | 32 | 13/19 | 11/21 | 77.1±2.3 | 78.5±2.6 | SP | PA |

Supplementary Table.1 the basic characteristics; Experimental group(EG): SuperPATH(SP) group; Control group(CG): conventional posterolateral/posterior approach(PA) group.

| Subgroup | Article  sizes | | Experimental groups’ sample sizes | Control groups’ sample sizes | Mean Difference%（95%CI） | Heterogeneity Test | | Z-test(P value） |
| --- | --- | --- | --- | --- | --- | --- | --- | --- |
|  |  |  |  |  |  | I^2^ | P |  |
| HHS | | | | | | | | |
| Preoperation | 21 | | 810 | 819 | 0.04 [-0.26 0.35] | 0.17 | 0.24 | 0.77 |
| One day after surgery | 3 | | 36 | 44 | 3.86 [-2.11, 9.832] | 0 | 0.91 | 0.2 |
| Three days after surgery | 4 | | 66 | 66 | 6.79 [1.41, 12.16] | 0.54 | 0.12 | 0.01 |
| One week after surgery | 8 | | 273 | 275 | 9.47 [6.21. 12.73] | 0.97 | <0.00001 | <0.00001 |
| Two weeks after surgery | 4 | | 76 | 80 | 1.80 [1.21, 2.40] | 0.89 | <0.00001 | <0.00001 |
| One month after surgery | 10 | | 438 | 427 | 7.17 [4.70, 9.64] | 0.98 | <0.00001 | <0.00001 |
| Three months after surgery | 22 | | 773 | 773 | 4.63 [3.28, 5.99] | 0.96 | <0.00001 | <0.00001 |
| Six  months after surgery | 20 | | 659 | 660 | 2.03 [1.14, 2.93] | 89 | <0.00001 | 0.0006 |
| One year after surgery | 8 | | 195 | 195 | 0.55 [0.14, 0.96] | 0 | 0.92 | 0.008 |
| VAS | | | | | | | | |
| Preoperation | 12 | | 423 | 423 | 0.01 [-0.09, 0.10] | 0 | 0.78 | 0.90 |
| One day after surgery | 6 | | 169 | 169 | -1.09 [-2.06, -0.12] | 0.98 | <0.00001 | 0.03 |
| One week after surgery | 8 | | 322 | 330 | -1.69 [-2.34, -1.04] | 0.99 | <0.00001 | <0.00001 |
| One month after surgery | 6 | | 271 | 277 | -0.91 [-1.59, -0.23] | 0.98 | <0.00001 | 0.009 |
| Three months after surgery | 8 | | 295 | 295 | -0.40 [-0.69, -0.12] | 0.87 | <0.00001 | 0.006 |
| Six  months after surgery | 6 | | 152 | 152 | -0.09 [-0.19, 0.00] | 0 | 0.78 | 0.06 |
| One year after surgery | 6 | | 168 | 126 | - 0.07 [-0.17, -0.02] | 0 | 0.93 | 0.12 |
| Barthel index | | | | | | | | |
| Preoperation | 4 | | 131 | 131 | 0.66 [-2.78, 4.10] | 0.76 | 0.005 | 0.71 |
| One week after surgery | 3 | | 106 | 106 | 6.44 [2.75, 10.13] | 0.57 | 0.1 | 0.0006 |
| Three months after surgery | 3 | | 106 | 106 | 6.17 [1.89, 10.44] | 0.78 | 0.01 | 0.005 |
| One year after surgery | 3 | | 81 | 81 | 4.22[-2.49, 10.93] | 0.94 | <0.00001 | 0.22 |
| Inflammatory indicators | | | | | | | | |
| CRP | | | | | | | | |
| Preoperation | | 3 | 81 | 79 | -0.11 [-0.61, 0.40] | 0 | 0.83 | 0.68 |
| Postoperation | | 3 | 81 | 79 | -12.28 [-29.71, 5.16] | 0.74 | 0.02 | 0.17 |
| ESR | | | | | | | | |
| Preoperation | | 3 | 81 | 79 | -1.04 [-3.96, 1.88] | 0 | 0.41 | 0.48 |
| Postoperation | | 3 | 81 | 79 | 1.36 [-1.81, 4.53] | 0 | 0.81 | 0.4 |
| CK | | | | | | | | |
| Preoperation | | 3 | 81 | 79 | -9.33 [-26.99, 8.33] | 0 | 0.66 | 0.3 |
| Postoperation | | 3 | 81 | 79 | -205.15 [-512.41, 102.12] | 0.92 | <0.00001 | 0.19 |

Supplementary Table.2 Experimental group(EG): SuperPATH group; Control group(CG): conventional posterolateral/posterior approach group; HHS: Harris Hip Score (HHS); VAS: Visual Analogue Score; CRP: C-Reactive Protein; ESR: Erythrocyte Sedimentation Rate; CK: Creatine Kinase.

| Subgroups | Article  sizes | Experimental groups’ sample sizes | | Control groups’ sample sizes | Risk Ratio%(95%CI） | Heterogeneity Test | | Z-test(P value） |
| --- | --- | --- | --- | --- | --- | --- | --- | --- |
|  |  |  |  |  |  | I^2^ | P |  |
| Complications | | | | | | | | |
| Postoperative dislocation of prosthetic joint | 19 | | 751 | 761 | 0.47 [0.21, 1.08] | 0 | 0.88 | 0.08 |
| Postoperative deep vein thrombosis of the lower limbs | 7 | | 283 | 289 | 0.43 [0.17, 1.11] | 0 | 0.73 | 0.08 |
| Postoperative sciatic nerve injury | 8 | | 309 | 308 | 0.60 [0.14, 2.46] | 0 | 0.71 | 0.48 |
| Postoperative periprosthetic infection | 6 | | 243 | 243 | 0.29 [0.07, 1.18] | 0 | 0.96 | 0.08 |
| Intraoperative blood transfusion cases | | | | | | | | |
| Intraoperative blood transfusion cases | 4 | | 120 | 120 | 0.29 [0.10, 0.84] | 0 | 0.59 | 0.02 |

Supplementary Table.3 Experimental group(EG): SuperPATH group; Control group(CG): conventional posterolateral/posterior approach group.
