# Supplementary material for: Comparison of clinical outcomes of supercapsular percutaneously-assisted approach total hip arthroplasty versus conventional posterior approach for total hip arthroplasty in adults: a systematic review and meta-analysis
Source: BMC Musculoskelet Disord. 2024 Jan 2;25:25. doi: 10.1186/s12891-023-07126-x (PMC10759432; doi:10.1186/s12891-023-07126-x)
Supplement: Supplementary file 3 — Additional file 3: Supplementary Figure 1. Supplementary Figure 2. Supplementary Figure 3. [file 12891_2023_7126_MOESM3_ESM.docx]

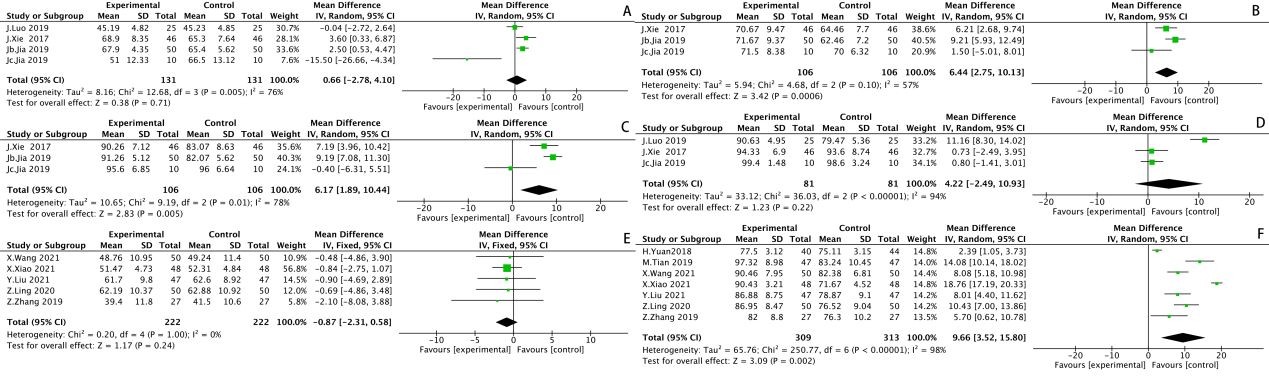


Supplementary Figure.1 Barthel Index (BI): Pre-operation (A); One week after surgery (B); Three months after surgery (C); One year after surgery (D); SF-36 score: Pre-operation (E); Post-operation (F).


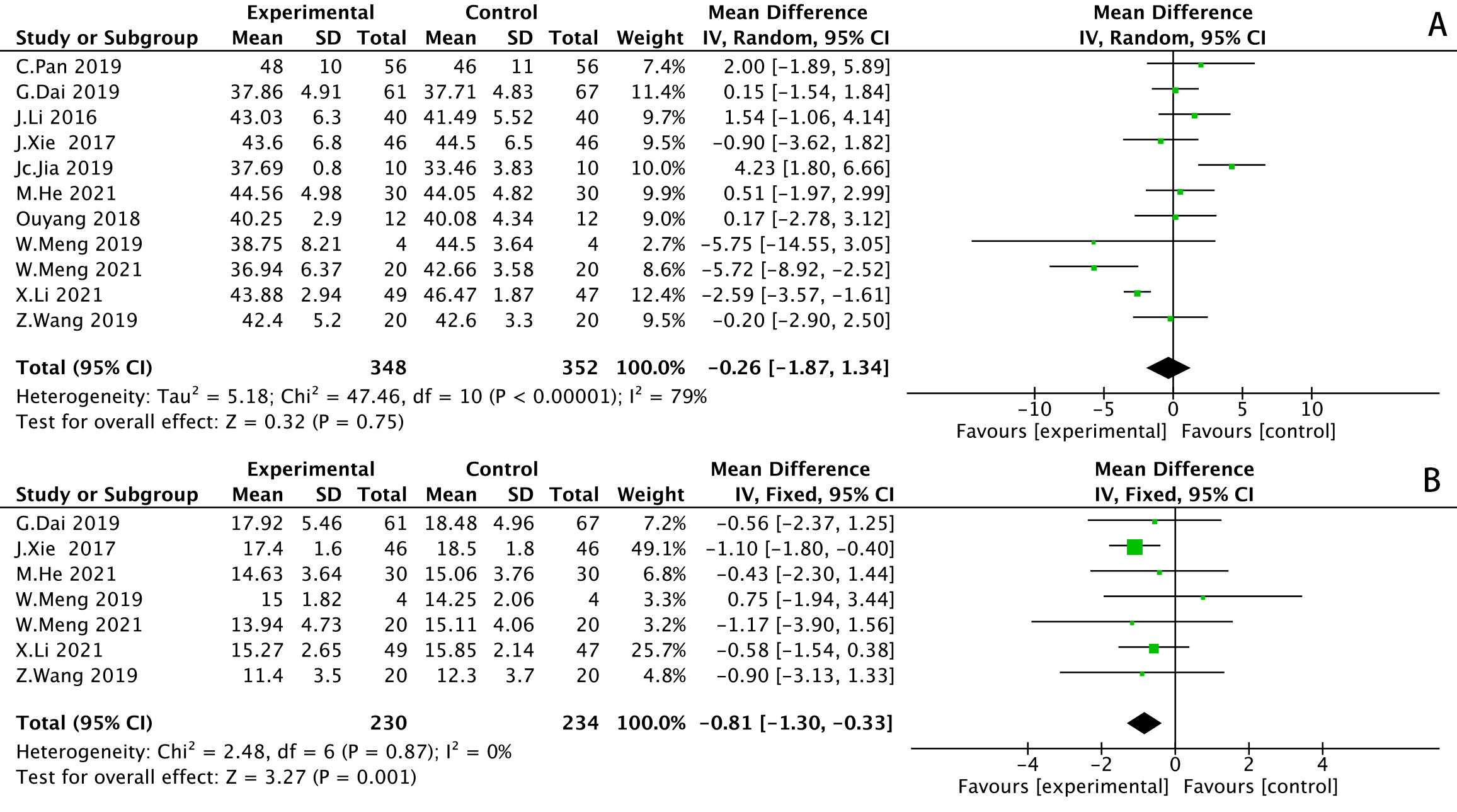


Supplementary Figure.2 Abduction angle (A); Anteversion angle (B).


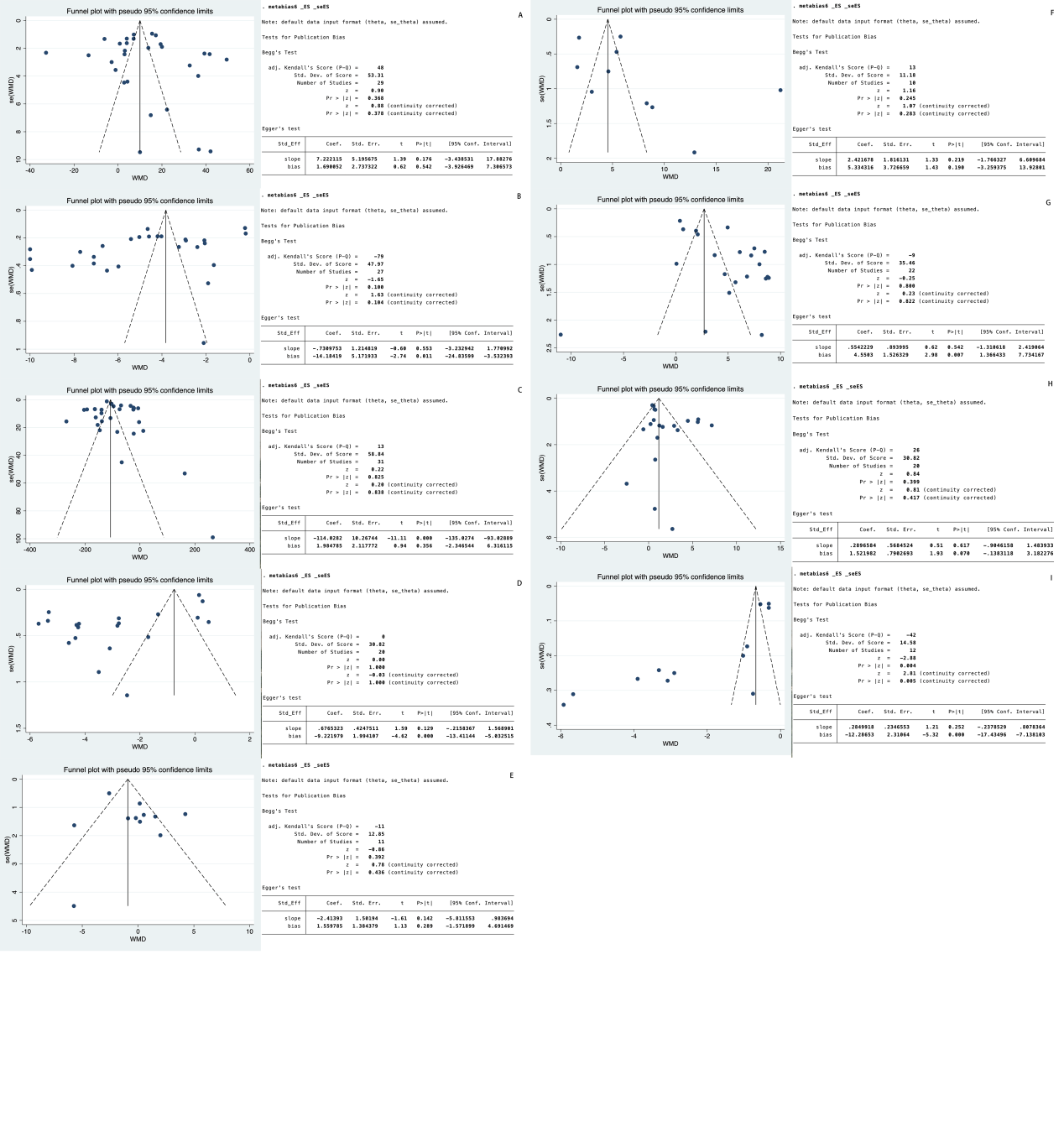


Supplementary Figure.3 Publication bias: Operation time (A); length of incision (B); Intraoperative blood loss (C); The lengths of hospital stay (D); Anteversion angle (E); One month after surgery HHS (F); Three months after surgery HHS (G); Six months after surgery HHS (H); Time to start activity postoperatively (I).
